# Supplementary material for: Decoupled transcript and protein concentrations ensure histone homeostasis in different nutrients
Source: EMBO J. 2024 Sep 13;43(21):5141–68. doi: 10.1038/s44318-024-00227-w (PMC11535423; doi:10.1038/s44318-024-00227-w)
Supplement: Supplementary file 1 — Appendix [file 44318_2024_227_MOESM1_ESM.pdf]

Appendix for:

Decoupled transcript and protein concentrations ensure histone homeostasis in different nutrients

Dimitra Chatzitheodoridou[1], Daniela Bureik[1], Francesco Padovani[1], Kalyan Varma Nadimpalli[1], Kurt M. Schmoller[1],

[1]Institute of Functional Epigenetics, Molecular Targets and Therapeutics Center, Helmholtz Zentrum München, 85764 Neuherberg, Germany *correspondence: kurt.schmoller@helmholtz-munich.de

Table of contents	Page
Appendix Figure S1	2
Appendix Figure S2	3
Appendix Table S1	5
Appendix Table S2	6
Appendix Table S3	9
Appendix Table S4	10

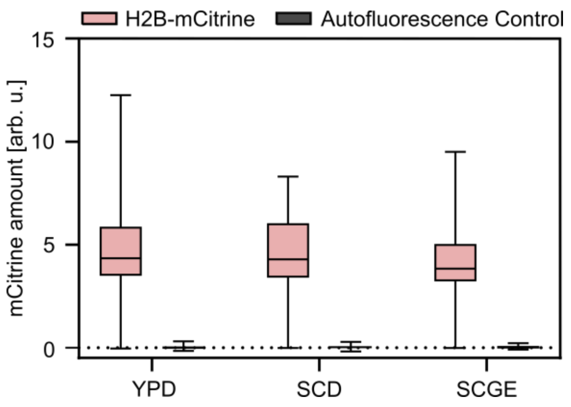

Appendix Figure S1. Autofluorescence control for the H2B-mCitrine strain in different growth media. Compared to H2B-mCitrine intensity, autofluorescence is negligible. Total mCitrine fluorescence intensity after background correction was measured in cells with mCitrine-tagged H2B (n_{YPD} = 492, n_{SCD} = 392 n_{SCGE} = 275) and untagged H2B (n_{YPD} = 227, n_{SCD} = 285 n_{SCGE} = 215). Box plots represent median and 25th and 75th percentiles, whiskers are extending to the minimum and maximum values.

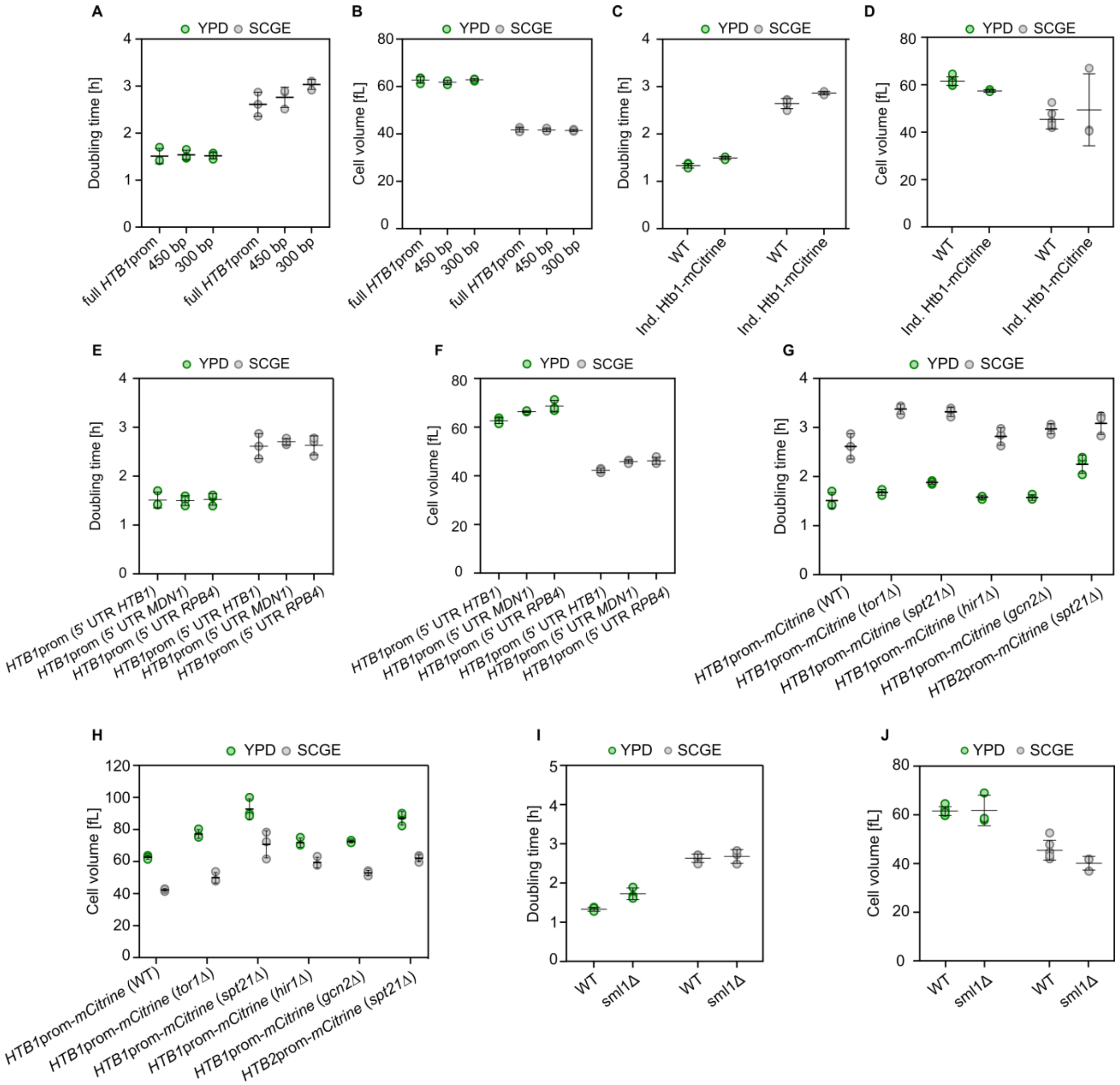

Appendix Figure S2. Growth phenotypes of strains used in figures 4, 5 and 6. (**A**) Nutrient-specific population doubling times and (**B**) mean volumes of cells carrying an additional copy of the full-length or truncated *HTB1* promoter driving the expression of mCitrine. Lines and error bars represent the means and standard deviations of n = 3 independent measurements shown as individual dots. (**C**) Nutrient-specific population doubling times and (**D**) mean cell volumes of wildtype cells and cells with β-estradiol inducible *HTB1-mCitrine*. Lines and error bars represent the means and standard deviations of n = 3-4 independent measurements shown as individual dots. (**E**) Nutrient-specific population doubling times and (**F**) mean volumes of cells expressing mCitrine with the *HTB1*, *MDN1* or *RPB4* 5' UTR. Lines and error bars represent the mean and standard deviation of n = 3 independent measurements shown as individual dots. (**G**) Population doubling times and (**H**) mean cell volumes of wildtype, *tor1Δ, spt21Δ, hir1Δ,* and *gcn2Δ* cells

expressing *HTB1*prom-mCitrine or *HTB2*prom-mCitrine in YPD and SCGE. Lines and error bars represent the means and standard deviations of n = 3 independent measurements shown as individual dots. (**I**) Population doubling times and (**J**) mean cell volumes of wildtype and *sml1Δ* cells growing in YPD and SCGE. Lines and error bars represent the means and standard deviations of n = 3-6 independent measurements shown as individual dots.

Appendix Table S1. Promoter sequences used for the experiments shown in Fig. 5, in which *mCitrine* mRNA is expressed from the *HTB1 promoter* with *HTB1, MDN1* or *RPB4* 5' UTR.

Gene feature	Position relative to start codon
***HTB1* promoter**	- 817 bp to -127 bp relative to *HTB1* ORF
***HTB1* 5' UTR**	127 bp upstream of *HTB1* ORF
***MDN1* 5' UTR**	150 bp upstream of *MDN1* ORF
***RPB4* 5' UTR**	125 bp upstream of *RPB4* ORF

Appendix Table S2. Strains used in this study.

Name	Genotype	Description	Origin	Figure
**ASY020-1**	*Mat α/a; ADE2/ADE2, URA3/ura3, leu2/LEU2*	Diploid wildtype strain	Anika Seel, Schmoller Lab	6
**CY14093**	*Mat α; sml1Δ::hphMX3*	Haploid *sml1Δ* strain	Christopher Bruhn	6, S2
**CY14098**	*Mat α; sml1Δ::hphMX3, rad53Δ::natMX6*	Haploid *sml1Δrad53Δ* strain	Christopher Bruhn	6
**CY15164**	*Mat α; sml1Δ::hphMX3, rad53Δ::natMX6, spt21Δ::kanMX6*	Haploid *sml1Δrad53Δ spt21Δ* strain	Christopher Bruhn	6
**DBY020-2**	*Mat α; ADE2, ura3::CglaTRP1-HTB1prom-mCitrine-ADH1term-URA3*	Haploid strain with additional copy of *HTB1* promoter driving mCitrine	This study	5
**DBY021-3**	*Mat α; ADE2, ura3::CglaTRP1-HTB2prom-mCitrine-ADH1term-URA3*	Haploid strain with additional copy of *HTB2* promoter driving mCitrine	This study	5
**DBY054-8**	*Mat a/α ; ADE2/ADE2, leu2-3/LEU2, URA3/ura3-1, htb1Δ::CglaTRP1/ /HTB1, htb2Δ::HIS3 /htb2Δ::NatMX6*	Diploid *HTB1/htb1Δ, htb2Δ/htb2Δ* strain	This study	6
**DBY064-1**	*Mat α; ADE2, ura3::CglaLEU2-HTB1prom-mCitrine-ADH1term-URA3*	Haploid strain with additional copy of *HTB1* promoter driving mCitrine	This study	4
**DBY065-2**	*Mat α ; ADE2, ura3::CglaLEU2-HTB1prom (mutated UAS3/UAS4)-mCitrine-ADH1term-URA3*	Haploid strain with additional copy of *HTB1* promoter, with mutated Spt10 binding sites in UAS3 and UAS4, driving mCitrine	This study	4
**DCY001-1**	*Mat α; ADE2, htb2::Htb2-linker-mCitrine-ADH1term-CglaTRP1, htb1::Htb1-linker-mCitrine-ADH1term-KlacURA3*	Haploid strain with *HTB1* and *HTB2* tagged with mCitrine	This study	1, EV1

**DCY002-2**	*Mat α; ADE2, ura3::CglaTRP1-HTB2prom-mCitrine-ADH11term-URA3,his3::ACT1prom-mKate2-ADH1term-HIS3*	Haploid strain with additional copy of *HTB2* promoter driving mCitrine and *ACT1* promoter driving mKate2	This study	3, EV3, EV4
**DCY008-8**	*Mat α; ADE2, ura3::CglaTRP1-HTB1prom-mCitrine-ADH1term-URA3,his3::ACT1prom-mKate2-ADH1term-HIS3*	Haploid strain with additional copy of *HTB1* promoter driving mCitrine and *ACT1* promoter driving mKate2	This study	3, 4, 5, EV3, EV4, EV5, S2
**DCY011-1**	*Mat α; ADE2, ura3::CglaTRP1-HTB1prom- MDN1 5'UTR-mCitrine-ADH1term-URA3*	Haploid strain with additional copy of *HTB1* promoter expressing mCitrine with *MDN1* 5' UTR	This study	5,S2
**DCY012-1**	*Mat α; ADE2, ura3::CglaTRP1-HTB1prom- RPB4 5'UTR-mCitrine-ADH1term-URA3*	Haploid strain with additional copy of *HTB1* promoter expressing mCitrine with *RPB4* 5' UTR	This study	5,S2
**DCY013-4**	*Mat α; ADE2, ura3::CglaTRP1-HTB1prom-mCitrine-ADH1term-URA3, his3::ACT1prom-mKate2-ADH1term-HIS3, tor1∆:: hphMX6*	Haploid *tor1∆* strain with additional copy of *HTB1* promoter driving mCitrine and *ACT1* promoter driving mKate2	This study	5, S2
**DCY14-3**	*Mat a; ADE2, LexApr-HTB1-mCitrine-ADH1term-URA3, his3::LexA-ER-AD-TF-HIS3*	Haploid strain expressing β-estradiol inducible Htb1-mCitrine	This study	EV5
**DCY15-1**	*Mat α; ADE2, ura3::CglaTRP1-HTB1prom-mCitrine-ADH1term-URA3, his3::ACT1prom-mKate2-ADH1term-HIS3, spt21∆:: LEU2*	Haploid *spt21∆* strain with additional copy of *HTB1* promoter driving mCitrine and *ACT1* promoter driving mKate2	This study	5, S2
**DCY16-1**	*Mat α; ADE2, ura3::CglaTRP1-HTB1prom-mCitrine-*	Haploid *hir1∆* strain with additional copy of *HTB1* promoter	This study	5, S2

	ADH1term-URA3, his3::ACT1prom-mKate2-ADH1term-HIS3, hir1Δ:: LEU2	driving mCitrine and *ACT1* promoter driving mKate2		
**DCY17-1**	*Mat α; ADE2, ura3::CglaTRP1-HTB1prom-mCitrine-ADH1term-URA3, his3::ACT1prom-mKate2-ADH1term-HIS3, gcn2Δ:: LEU2*	Haploid *gcn2Δ* strain with additional copy of *HTB1* promoter driving mCitrine and *ACT1* promoter driving mKate2	This study	5, S2
**JE621**	*Mat α, ADE2, TRP1, LEU2, his3::LexO transcription factor-HIS3, cdc20::natMX-lexOPr-Cdc20*	Haploid strain expressing β-estradiol inducible Cdc20	Jennifer Ewald Lab	2, EV2
**KCY021-1**	*Mat α; ADE2, ura3::CglaTRP1-300bpHTB1prom-mCitrine-ADH1term-URA3*	Haploid strain with additional 300 bp truncation of *HTB1* promoter driving mCitrine	Kora-Lee Claude, Schmoller Lab	4, S2
**KCY022-1**	*Mat α; ADE2, ura3::CglaTRP1-450bpHTB1prom-mCitrine-ADH1term-URA3*	Haploid strain with additional 450 bp truncation of *HTB1* promoter driving mCitrine	Kora-Lee Claude, Schmoller Lab	4, S2
**KSY229-1**	*Mat α; ADE2, ura3::CglaTRP1-ACT1prom-mCitrine-ADH1term-URA3*	Haploid strain with additional copy of *ACT1* promoter driving mCitrine	This study	3, EV3, EV4
**MMY116-2C**	*Mat α; ADE2*	Haploid wildtype strain	Skotheim lab stock	1, 2, 3, EV1, EV2, EV3, EV4, S1, S2

Appendix Table S3. Sequences of qPCR primers used in this study.

Gene	qPCR primer direction	qPCR primer sequence (5‘-3‘)
**HTB1**	forward	TACACACATACAATGTCTGCTAAAG
	reverse	AGTGTCAGGGTGAGTTTGCTT
**HTB2**	forward	CCTCTGCCGCCGAAAAGAAA
	reverse	TCTTACCATCGACGGAGGTTG
**HTA1**	forward	GTTGCCAAAGAAGTCTGCCA
	reverse	CAGTTTAGTTCCTTCCGCCTT
**HTA2**	forward	TCGCCCAAGGTGGTGTTTT
	reverse	TGATTTGCTTTGTTTCTTTTCAACT
**HHF1**	forward	TACACCGAACACGCCAAGAG
	reverse	TTGCTTGTTGTTACCGTTTTCTT
**HHF2**	forward	ACGAAGAAGTCAGAGCCGTC
	reverse	ACCGATTGTTTAACCACCGATTG
**HHT1**	forward	CAATCTTCTGCCATCGGTGC
	reverse	ACTGATGACAATCAACAAACTATGA
**HHT2**	forward	AGCAAACACTCCACAATGGC
	reverse	CAAGGCAACAGTACCTGGCT
**ACT1**	forward	AGTTGCCCCAGAAGAACACC
	reverse	GGACAAAACGGCTTGGATGG
**MDN1**	forward	CATCAACAAACCTGACCAACTAATCC
	reverse	CATCAAGGTTTTCCAAAGTGGGC
**mCitrine**	forward	GAGCTGAAGGGCATCGACTT
	reverse	TTCTGCTTGTCGGCCATGAT
**RDN18**	forward	AACTCACCAGGTCCAGACACAATAAGG
	reverse	AAGGTCTCGTTCGTTATCGCAATTAAGC

Appendix Table S4. Sequences of qPCR primers used for mRNA stability measurements shown in figure 2B.

Gene	qPCR primer direction	qPCR primer sequence (5‘-3‘)
**HTB1**	forward	TGGCTGCGTATAACAAGAAGTCT
	reverse	CCAAAGGAAGTGATTTCATTATGC
**HTB2**	forward	TGCTCTATACTCAAACCAACAACA
	reverse	ATCTCTTCTTACCATCGACGGA
**ACT1**	forward	TATGTGTAAAGCCGGTTTTGC
	reverse	GACAATAC CGTGTTCAATTGGG
