# Supplementary figures and images for: Decoupled transcript and protein concentrations ensure histone homeostasis in different nutrients
Source: EMBO J. 2024 Sep 13;43(21):5141–68. doi: 10.1038/s44318-024-00227-w (PMC11535423; doi:10.1038/s44318-024-00227-w)

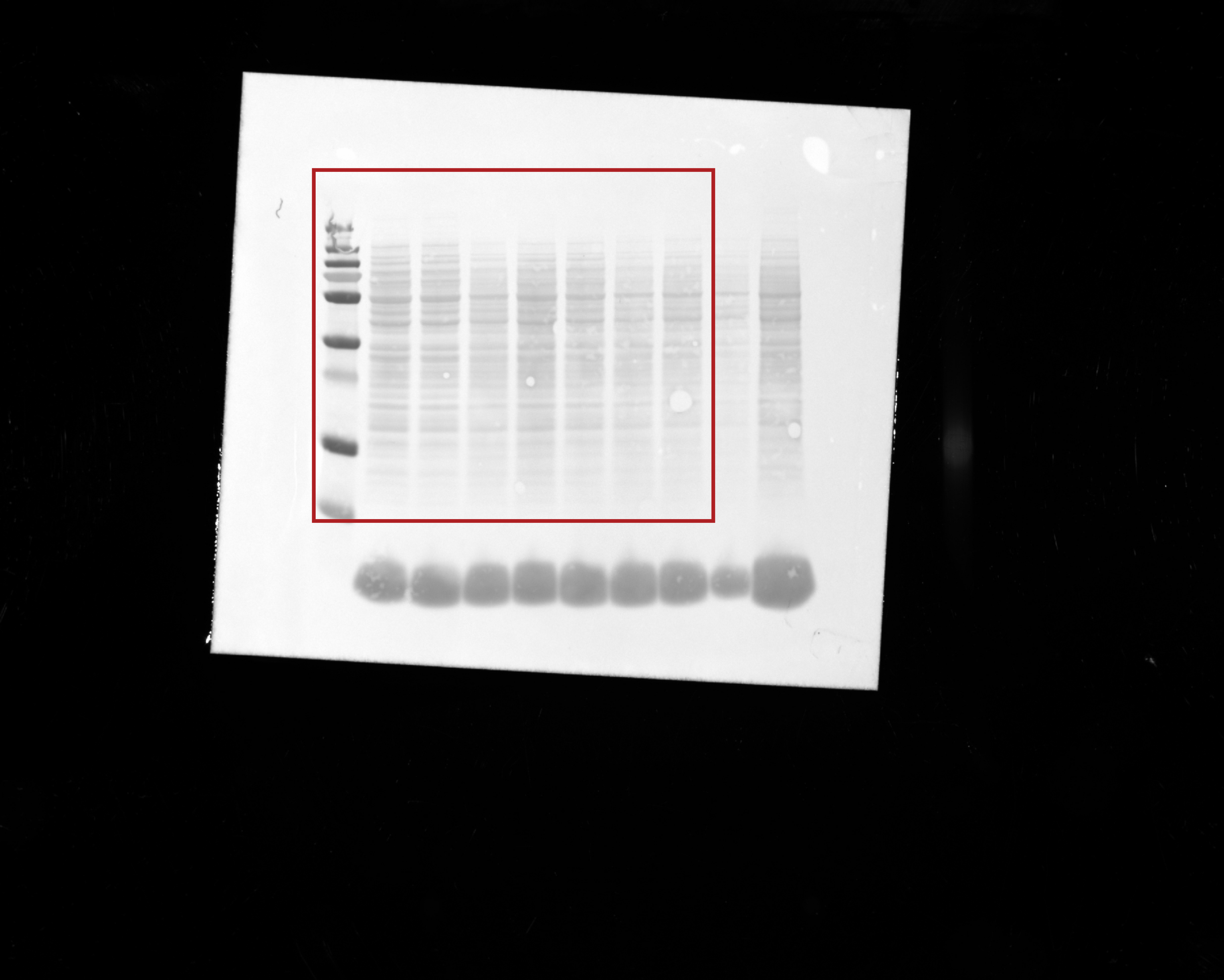

Supplement: Supplementary file 3 — Source data Fig. 1 [file 44318_2024_227_MOESM3_ESM.zip › Figure 1/1D/EV1B/Blot_cropped.tiff]

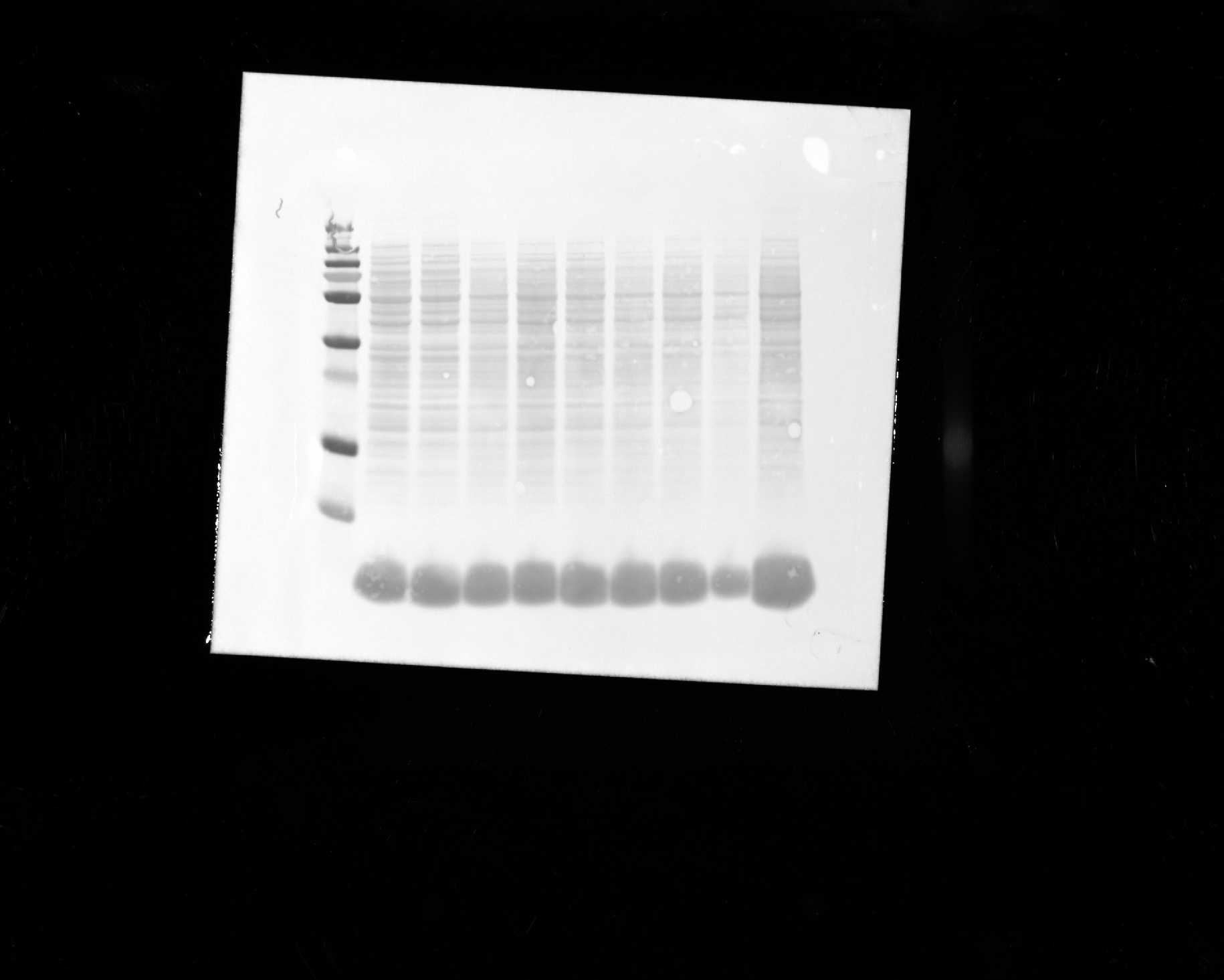

Supplement: Supplementary file 3 — Source data Fig. 1 [file 44318_2024_227_MOESM3_ESM.zip › Figure 1/1D/EV1B/Ponceau Stain.jpg]

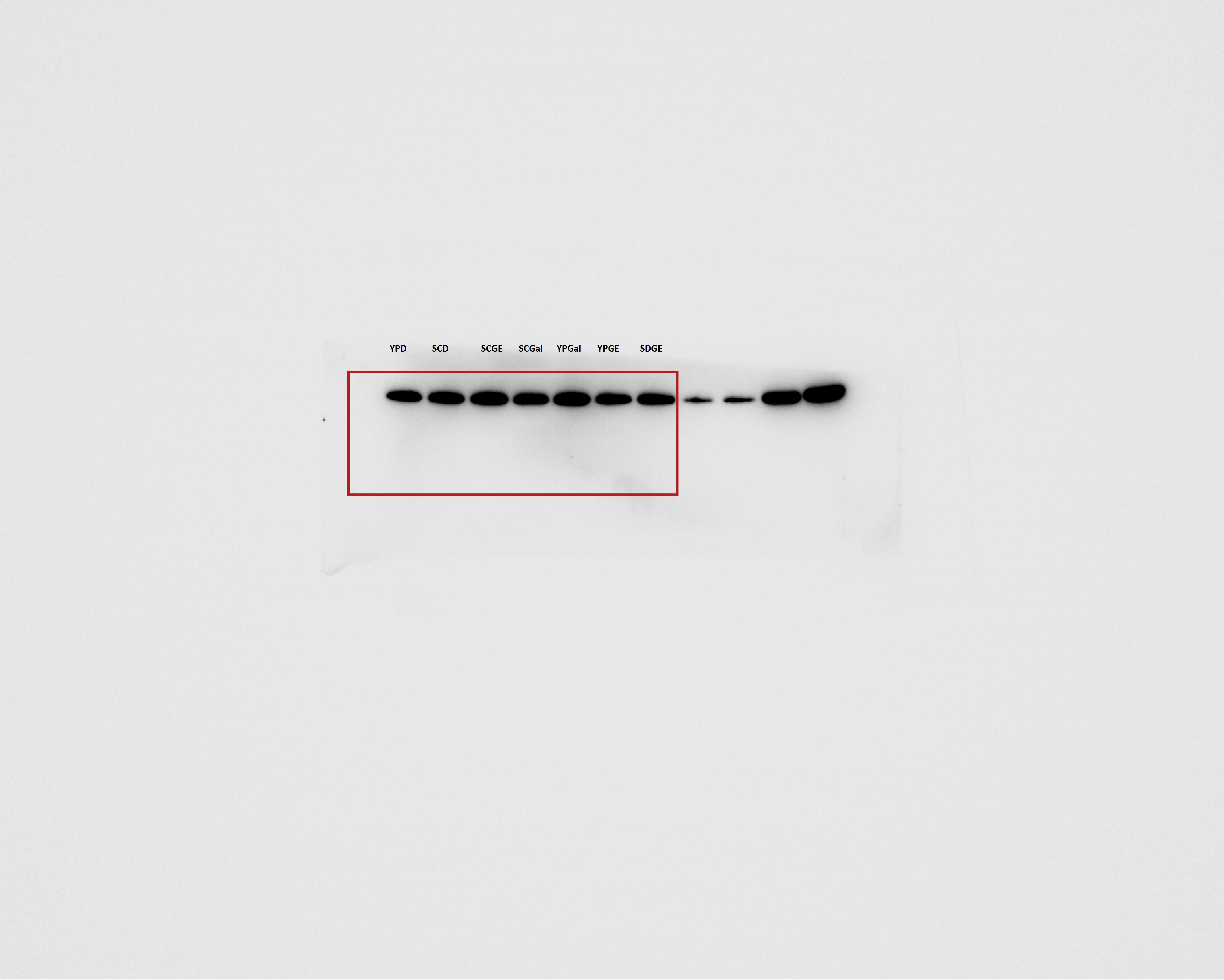

Supplement: Supplementary file 3 — Source data Fig. 1 [file 44318_2024_227_MOESM3_ESM.zip › Figure 1/1E/Western blot images anti-H2B/anti-H2B bands.jpg]

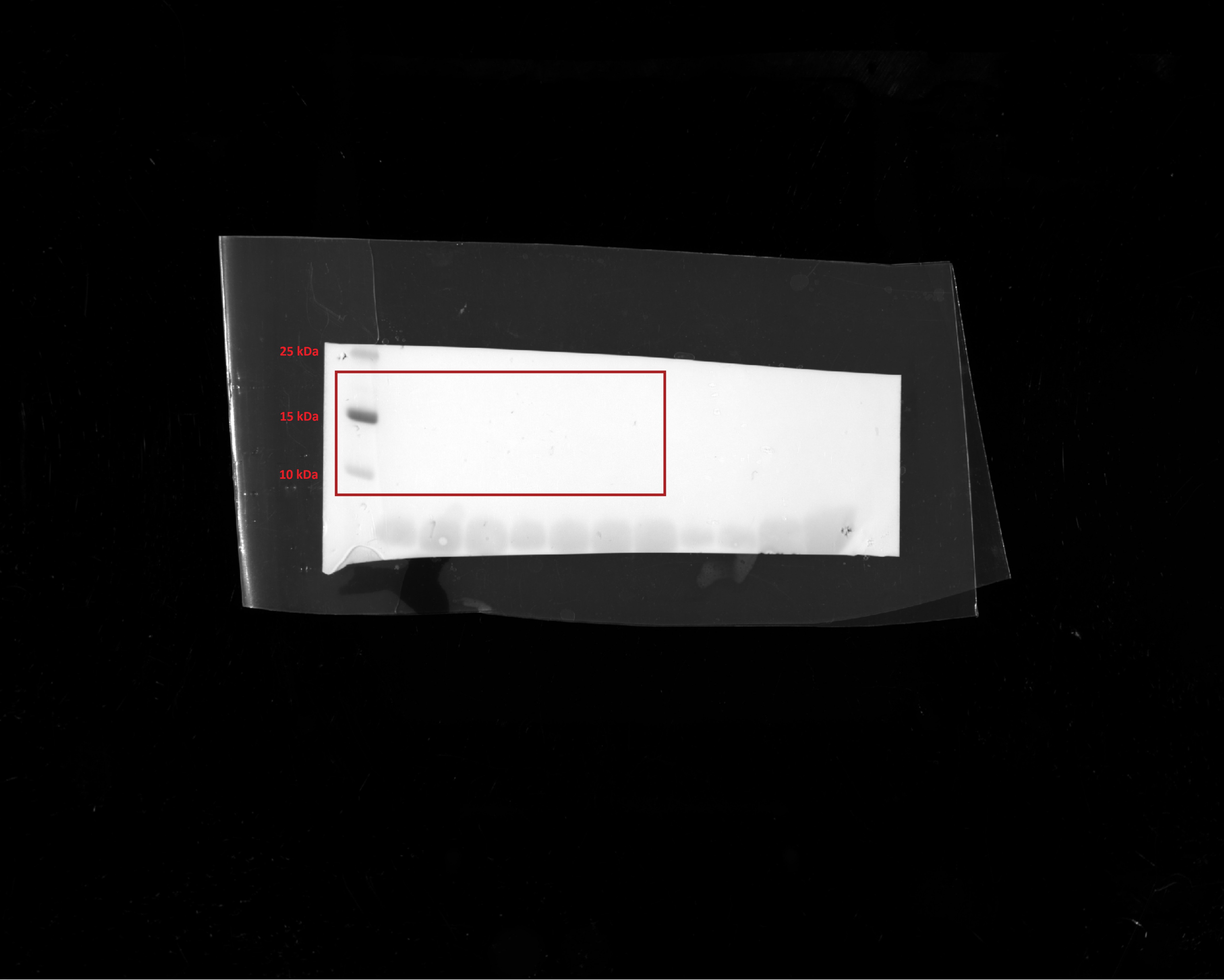

Supplement: Supplementary file 3 — Source data Fig. 1 [file 44318_2024_227_MOESM3_ESM.zip › Figure 1/1E/Western blot images anti-H2B/anti-H2B ladder.jpg]

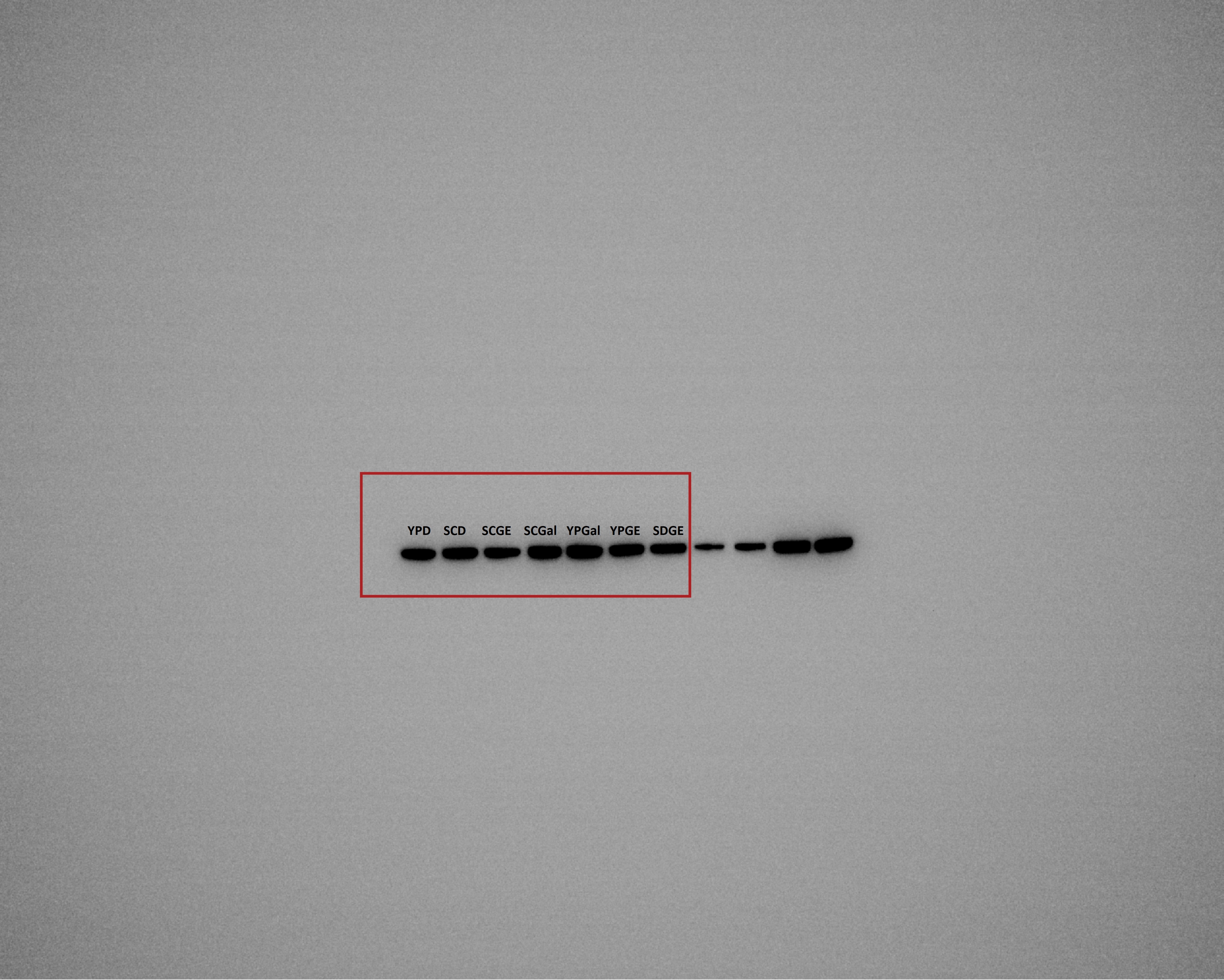

Supplement: Supplementary file 3 — Source data Fig. 1 [file 44318_2024_227_MOESM3_ESM.zip › Figure 1/1F/Western blot images anti-β actin/anti-ACT1 bands.jpg]

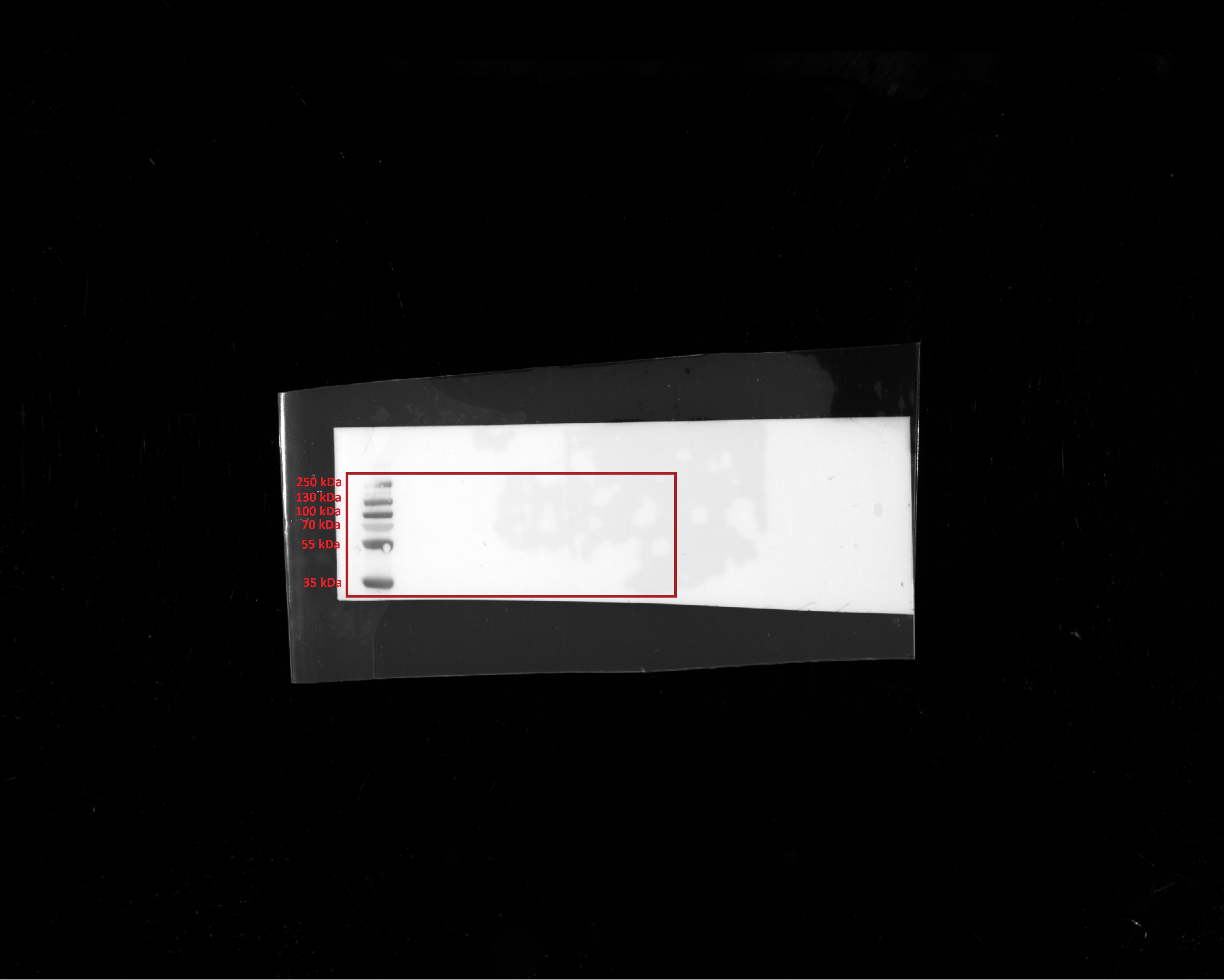

Supplement: Supplementary file 3 — Source data Fig. 1 [file 44318_2024_227_MOESM3_ESM.zip › Figure 1/1F/Western blot images anti-β actin/anti-ACT1 ladder.jpg]

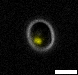

Supplement: Supplementary file 3 — Source data Fig. 1 [file 44318_2024_227_MOESM3_ESM.zip › Figure 1/1G/Microscopy images/SCD/20020302_DCY001_1_SCD_19.png]

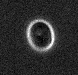

Supplement: Supplementary file 3 — Source data Fig. 1 [file 44318_2024_227_MOESM3_ESM.zip › Figure 1/1G/Microscopy images/SCD/20020302_DCY001_1_SCD_19.tif]

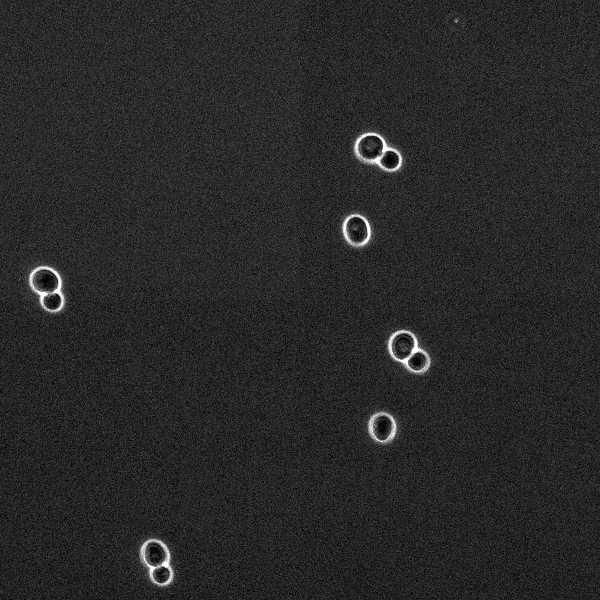

Supplement: Supplementary file 3 — Source data Fig. 1 [file 44318_2024_227_MOESM3_ESM.zip › Figure 1/1G/Microscopy images/SCD/20020302_DCY001_1_SCD_19_cropped.tif]

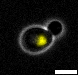

Supplement: Supplementary file 3 — Source data Fig. 1 [file 44318_2024_227_MOESM3_ESM.zip › Figure 1/1G/Microscopy images/SCD/20020302_DCY001_1_SCD_21.png]

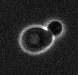

Supplement: Supplementary file 3 — Source data Fig. 1 [file 44318_2024_227_MOESM3_ESM.zip › Figure 1/1G/Microscopy images/SCD/20020302_DCY001_1_SCD_21.tif]

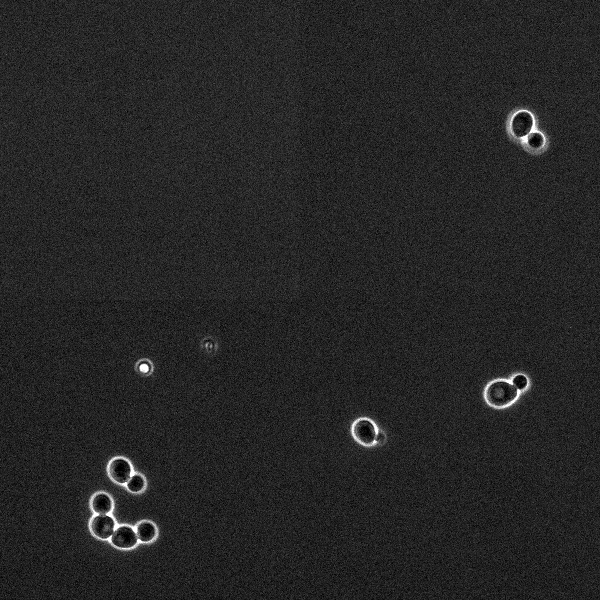

Supplement: Supplementary file 3 — Source data Fig. 1 [file 44318_2024_227_MOESM3_ESM.zip › Figure 1/1G/Microscopy images/SCD/20020302_DCY001_1_SCD_21_cropped.tif]

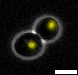

Supplement: Supplementary file 3 — Source data Fig. 1 [file 44318_2024_227_MOESM3_ESM.zip › Figure 1/1G/Microscopy images/SCD/20020302_DCY001_1_SCD_24.png]

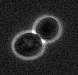

Supplement: Supplementary file 3 — Source data Fig. 1 [file 44318_2024_227_MOESM3_ESM.zip › Figure 1/1G/Microscopy images/SCD/20020302_DCY001_1_SCD_24.tif]

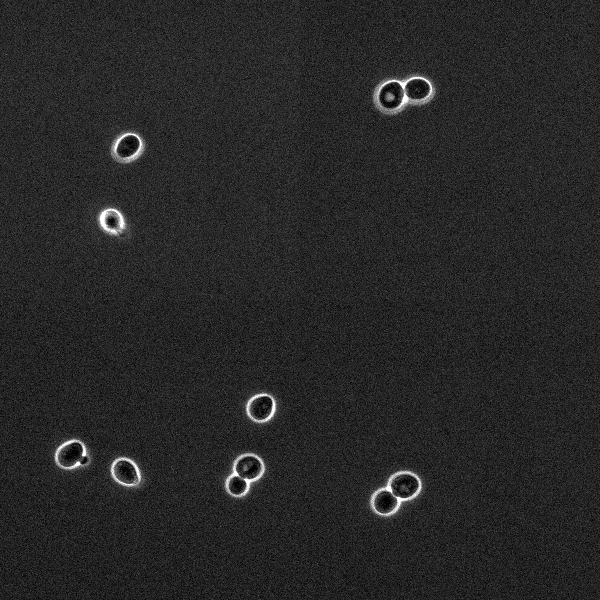

Supplement: Supplementary file 3 — Source data Fig. 1 [file 44318_2024_227_MOESM3_ESM.zip › Figure 1/1G/Microscopy images/SCD/20020302_DCY001_1_SCD_24_cropped.tif]

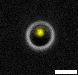

Supplement: Supplementary file 3 — Source data Fig. 1 [file 44318_2024_227_MOESM3_ESM.zip › Figure 1/1G/Microscopy images/SCGE/20220303_DCY1_SCGE_10.png]

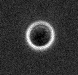

Supplement: Supplementary file 3 — Source data Fig. 1 [file 44318_2024_227_MOESM3_ESM.zip › Figure 1/1G/Microscopy images/SCGE/20220303_DCY1_SCGE_10.tif]

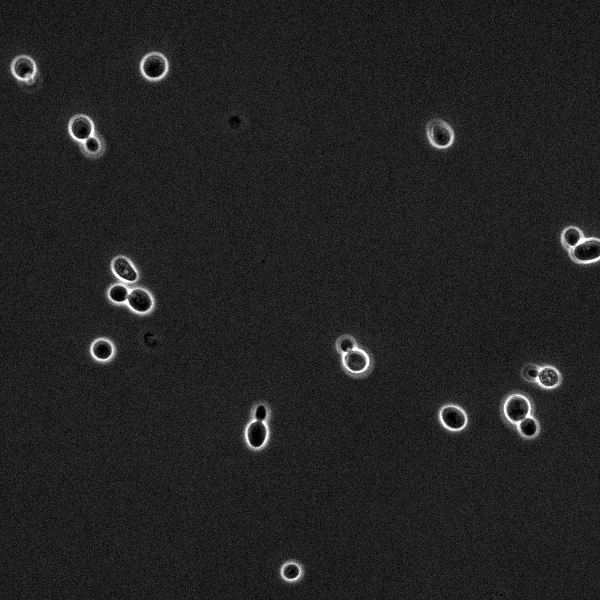

Supplement: Supplementary file 3 — Source data Fig. 1 [file 44318_2024_227_MOESM3_ESM.zip › Figure 1/1G/Microscopy images/SCGE/20220303_DCY1_SCGE_10_cropped.tif]

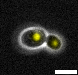

Supplement: Supplementary file 3 — Source data Fig. 1 [file 44318_2024_227_MOESM3_ESM.zip › Figure 1/1G/Microscopy images/SCGE/20220303_DCY1_SCGE_19.png]

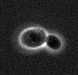

Supplement: Supplementary file 3 — Source data Fig. 1 [file 44318_2024_227_MOESM3_ESM.zip › Figure 1/1G/Microscopy images/SCGE/20220303_DCY1_SCGE_19.tif]

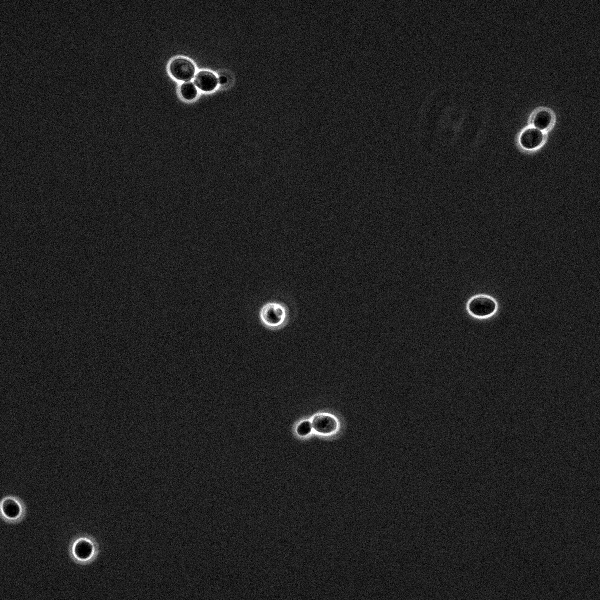

Supplement: Supplementary file 3 — Source data Fig. 1 [file 44318_2024_227_MOESM3_ESM.zip › Figure 1/1G/Microscopy images/SCGE/20220303_DCY1_SCGE_19_cropped.tif]

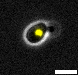

Supplement: Supplementary file 3 — Source data Fig. 1 [file 44318_2024_227_MOESM3_ESM.zip › Figure 1/1G/Microscopy images/SCGE/20220303_DCY1_SCGE_2.png]

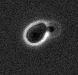

Supplement: Supplementary file 3 — Source data Fig. 1 [file 44318_2024_227_MOESM3_ESM.zip › Figure 1/1G/Microscopy images/SCGE/20220303_DCY1_SCGE_2.tif]

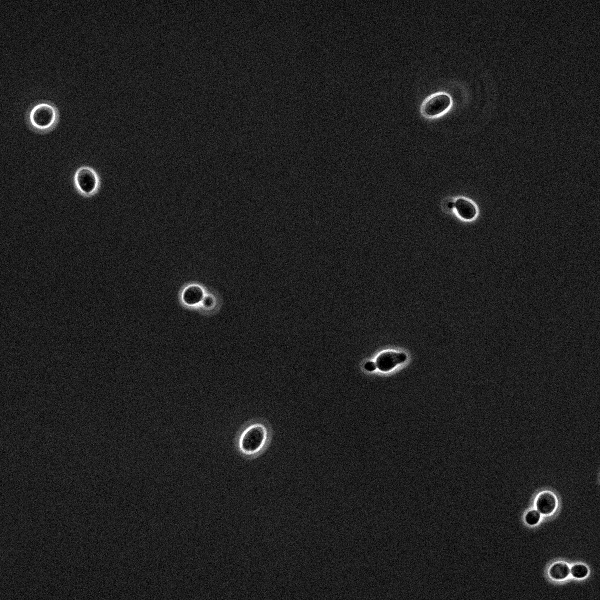

Supplement: Supplementary file 3 — Source data Fig. 1 [file 44318_2024_227_MOESM3_ESM.zip › Figure 1/1G/Microscopy images/SCGE/20220303_DCY1_SCGE_2_cropped.tif]

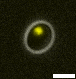

Supplement: Supplementary file 3 — Source data Fig. 1 [file 44318_2024_227_MOESM3_ESM.zip › Figure 1/1G/Microscopy images/YPD/20220302_DCY1_1_YPD_10.png]

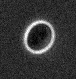

Supplement: Supplementary file 3 — Source data Fig. 1 [file 44318_2024_227_MOESM3_ESM.zip › Figure 1/1G/Microscopy images/YPD/20220302_DCY1_1_YPD_10.tif]

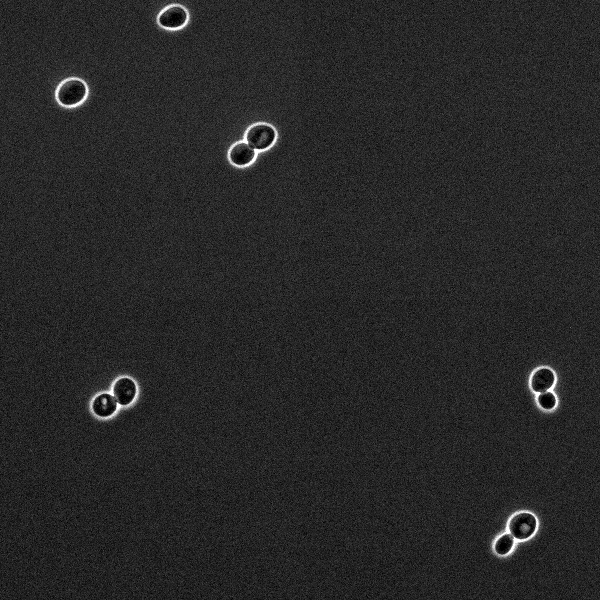

Supplement: Supplementary file 3 — Source data Fig. 1 [file 44318_2024_227_MOESM3_ESM.zip › Figure 1/1G/Microscopy images/YPD/20220302_DCY1_1_YPD_10_cropped.tif]

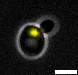

Supplement: Supplementary file 3 — Source data Fig. 1 [file 44318_2024_227_MOESM3_ESM.zip › Figure 1/1G/Microscopy images/YPD/20220302_DCY1_1_YPD_14.png]

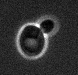

Supplement: Supplementary file 3 — Source data Fig. 1 [file 44318_2024_227_MOESM3_ESM.zip › Figure 1/1G/Microscopy images/YPD/20220302_DCY1_1_YPD_14.tif]

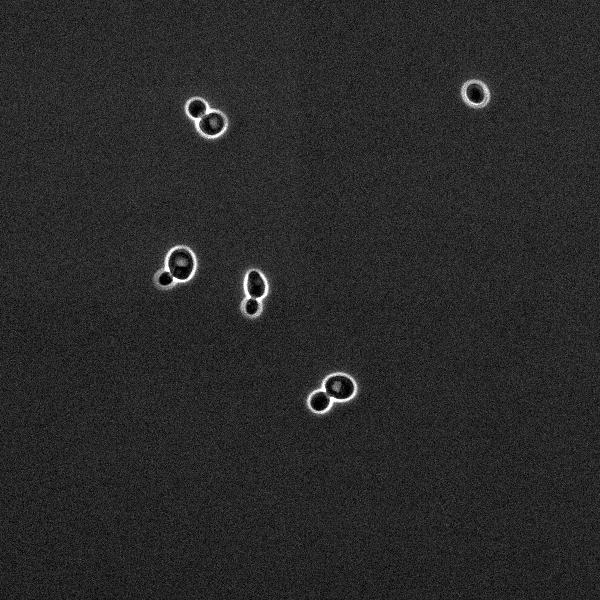

Supplement: Supplementary file 3 — Source data Fig. 1 [file 44318_2024_227_MOESM3_ESM.zip › Figure 1/1G/Microscopy images/YPD/20220302_DCY1_1_YPD_14_cropped.tif]

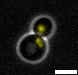

Supplement: Supplementary file 3 — Source data Fig. 1 [file 44318_2024_227_MOESM3_ESM.zip › Figure 1/1G/Microscopy images/YPD/20220302_DCY1_1_YPD_7.png]

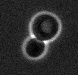

Supplement: Supplementary file 3 — Source data Fig. 1 [file 44318_2024_227_MOESM3_ESM.zip › Figure 1/1G/Microscopy images/YPD/20220302_DCY1_1_YPD_7.tif]

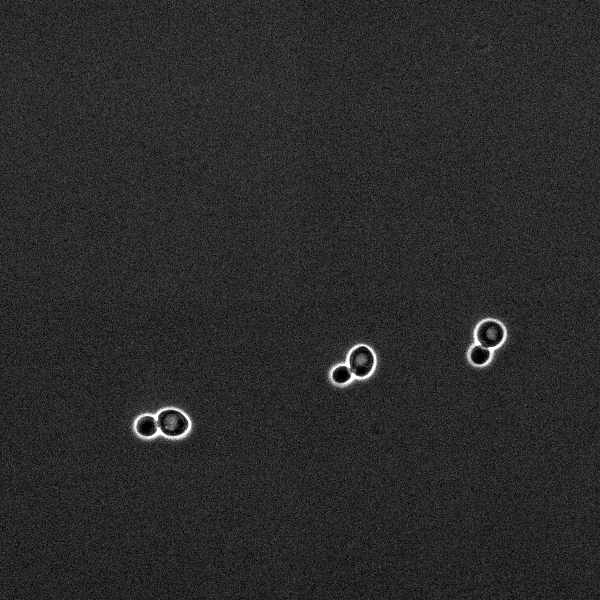

Supplement: Supplementary file 3 — Source data Fig. 1 [file 44318_2024_227_MOESM3_ESM.zip › Figure 1/1G/Microscopy images/YPD/20220302_DCY1_1_YPD_7_cropped.tif]

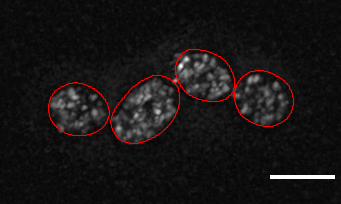

Supplement: Supplementary file 5 — Source data Fig. 3 [file 44318_2024_227_MOESM5_ESM.zip › Figure 3/3C/microscopy images/MMY116_2c_ACT1_MDN1_10_s02_ACT1_preprocessed__MAX_ACT1_gray.png]

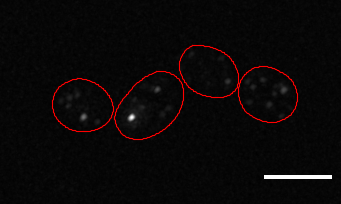

Supplement: Supplementary file 5 — Source data Fig. 3 [file 44318_2024_227_MOESM5_ESM.zip › Figure 3/3C/microscopy images/MMY116_2c_ACT1_MDN1_10_s02_MDN1_preprocessed__MAX_MDN1_gray.png]

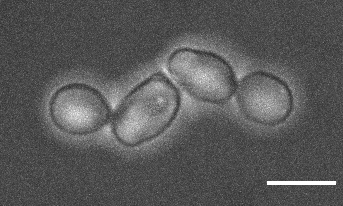

Supplement: Supplementary file 5 — Source data Fig. 3 [file 44318_2024_227_MOESM5_ESM.zip › Figure 3/3C/microscopy images/MMY_ACT1_MDN1_YPD_BF_noscale.png]

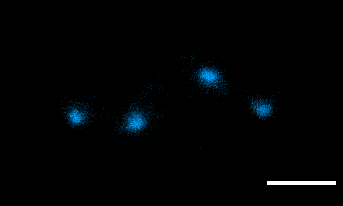

Supplement: Supplementary file 5 — Source data Fig. 3 [file 44318_2024_227_MOESM5_ESM.zip › Figure 3/3C/microscopy images/MMY_ACT1_MDN1_YPD_DAPI_noscale.png]

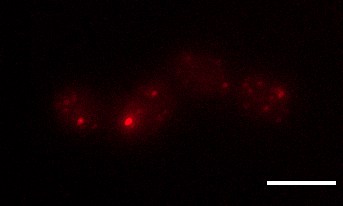

Supplement: Supplementary file 5 — Source data Fig. 3 [file 44318_2024_227_MOESM5_ESM.zip › Figure 3/3C/microscopy images/MMY_ACT1_MDN1_YPD_RED_noscale.png]

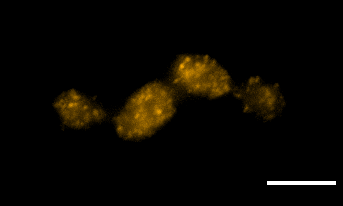

Supplement: Supplementary file 5 — Source data Fig. 3 [file 44318_2024_227_MOESM5_ESM.zip › Figure 3/3C/microscopy images/MMY_ACT1_MDN1_YPD_Yellow_noscale.png]

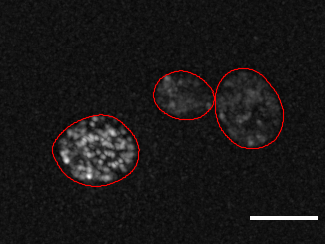

Supplement: Supplementary file 5 — Source data Fig. 3 [file 44318_2024_227_MOESM5_ESM.zip › Figure 3/3E/microscopy images/DCY008_8_YPD_9_s18_mCitr_preprocessed__MAX_mCitr_gray.png]

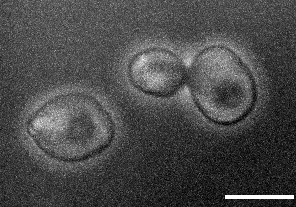

Supplement: Supplementary file 5 — Source data Fig. 3 [file 44318_2024_227_MOESM5_ESM.zip › Figure 3/3E/microscopy images/MAX_DCY008_8_YPD_BF.png]

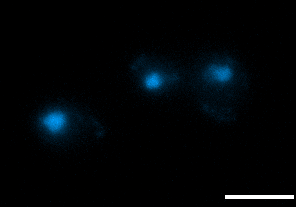

Supplement: Supplementary file 5 — Source data Fig. 3 [file 44318_2024_227_MOESM5_ESM.zip › Figure 3/3E/microscopy images/MAX_DCY008_8_YPD_DAPI.png]

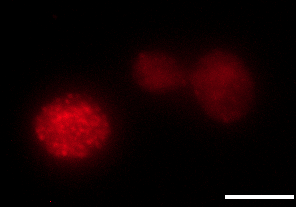

Supplement: Supplementary file 5 — Source data Fig. 3 [file 44318_2024_227_MOESM5_ESM.zip › Figure 3/3E/microscopy images/MAX_DCY008_8_YPD_red.png]
