# Supplementary material for: Decoupled transcript and protein concentrations ensure histone homeostasis in different nutrients
Source: EMBO J. 2024 Sep 13;43(21):5141–68. doi: 10.1038/s44318-024-00227-w (PMC11535423; doi:10.1038/s44318-024-00227-w)
Supplement: Supplementary file 9 — Expanded View Figures [file 44318_2024_227_MOESM9_ESM.pdf]

Expanded View Figures

Figure EV1. Analysis of nutrient-dependent histone H2B amounts at steady state and during nutrient upshift.

(**A**) Doubling times were calculated from growth curves of wild-type cells growing in different nutrient conditions as a function of the nutrient-dependent cell volumes measured with a Coulter counter. Mean and standard deviation of $n = 4$–6 replicates are shown; line shows linear fit. (**B**) Representative western blot membrane stained with Ponceau S for quantification of total proteins. (**C**) Total protein content, extracted from equal numbers of cells in different growth media, is normalized on YPD and plotted against the nutrient-dependent cell volume measured with a Coulter counter. Mean and standard deviation of $n = 4$–7 replicates are shown; line shows linear fit. (**D**) Single-cell expression profiles of H2B-mCitrine (Htb1 and Htb2 tagged) corresponding to Fig. 1I ($n_{YPD} = 87$, $n_{SCD} = 83$, $n_{SCGE} = 55$). (**E**) Quantification of the duration of the H2B-mCitrine production phase in the different nutrients. Box plots represent the median and 25th and 75th percentiles, whiskers indicate the 5th and 95th percentiles, and symbols show outliers. The H2B-mCitrine production phase was determined as described in Methods and Materials; $^{***}P_{YPD\text{-}SCGE} = 0.0003$; $^{**}p_{SCD\text{-}SCGE} = 0.0023$. (**F**) Total H2B-mCitrine amounts in G1 were quantified using flow cytometry in different growth media. Lines represent the mean of $n = 2$ independent replicates, each shown as an individual dot. (**G**) Nutrient-dependent cell cycle distributions (percentage of cells in G1-, S-, and G2/M-phase) of cells with *mCitrine*-tagged H2B, determined with flow cytometry using the H2B fluorescence intensity. The bar graphs represent the mean of $n = 2$ independent replicates, each shown as an individual dot. (**H**) Characterization of H2B-mCitrine strain in different growth media. Doubling times were calculated from growth curves of cells with *mCitrine*-tagged *HTB1* and *HTB2*, growing in different nutrient conditions. Lines and error bars represent the means and standard deviations of $n = 3$ independent measurements, each shown as an individual dot. (**I**) Nutrient-specific mean cell volumes of cells with *mCitrine*-tagged *HTB1* and *HTB2* were measured with a Coulter counter. Lines represent the mean of $n = 2$ independent measurements shown as individual dots. (**J**) RT-qPCR was used to measure the mRNA concentrations of *HTB1* and *HTB2*, as well as the control gene *ACT1* in wild-type cells and cells with *mCitrine*-tagged *HTB1* and *HTB2*. mRNA concentrations were normalized on *RDN18* and are shown as mean fold changes compared to the wild-type strain. Error bars indicate standard errors of at least three independent biological replicates. (**K**) Cells maintain constant histone protein amounts during nutrient upshift. To study how cells adjust histone production in response to a dynamic nutrient switch, cells with mCitrine-tagged H2B were grown in a microfluidic plate on SCGE for 5 h before being shifted to YPD medium (time = 0). Total mCitrine intensity in newborn daughter cells was measured over the course of the experiment using time-lapse microscopy ($n_{SCGE} = 73$, $n_{YPD} = 519$, $n_{background} = 565$). Lines connect binned means with error bars representing standard errors.

 © The Author(s)

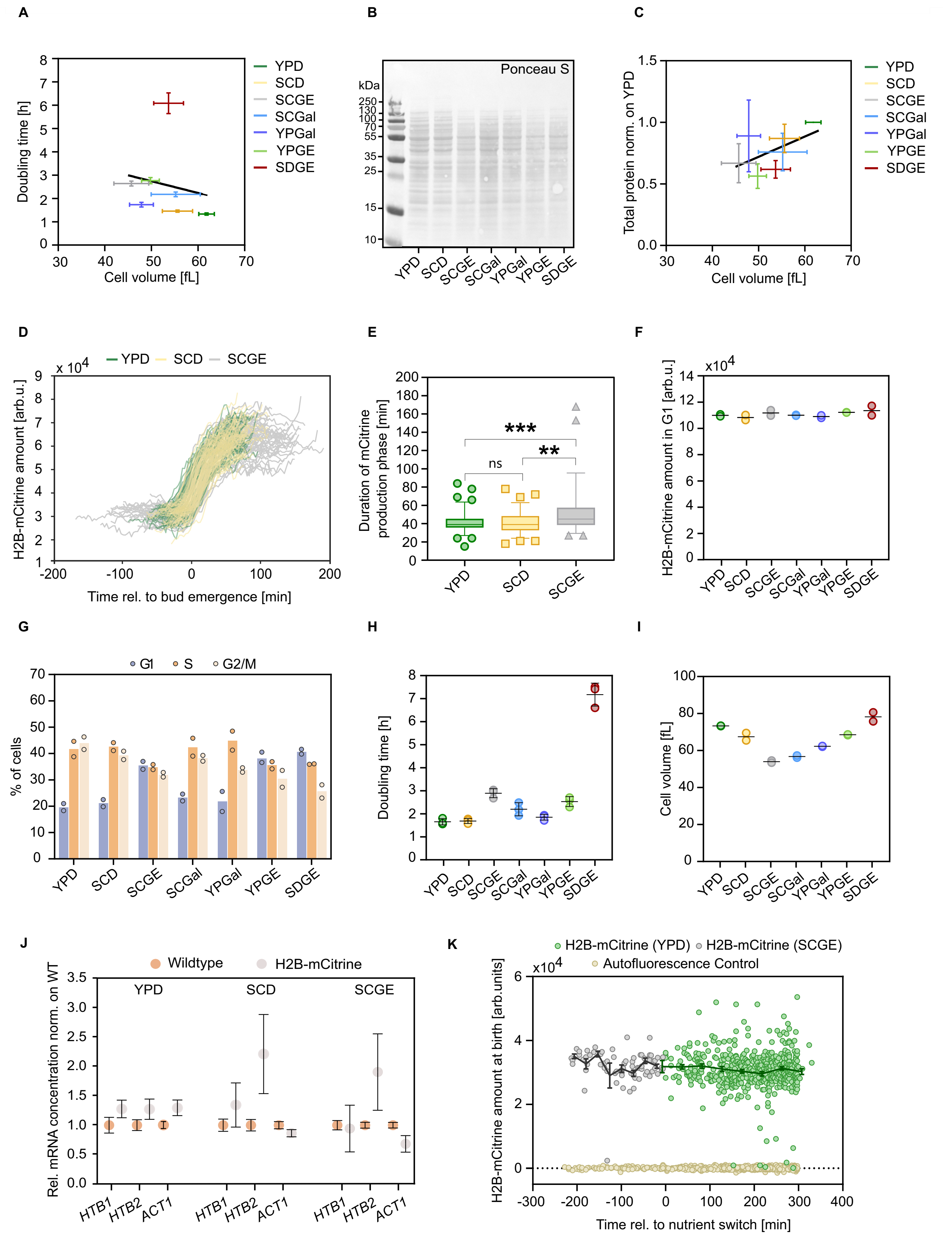
A
Doubling time [h]
Cell volume [fL]
YPD
SCD
SCGE
SCGal
YPGal
YPGE
SDGE
B
Ponceau S
kDa
250
130
100
70
55
35
25
15
10
YPD SCD SCGE SCGal YPGal YPGE SDGE
C
Total protein norm. on YPD
Cell volume [fL]
D
x 10^4
YPD SCD SCGE
H2B-mCitrine amount [arb.u.]
Time rel. to bud emergence [min]
E
Duration of mCitrine production phase [min]
***
**
ns
YPD SCD SCGE
F
x10^4
H2B-mCitrine amount in G1 [arb.u.]
G
G1 S G2/M
% of cells
H
Doubling time [h]
I
Cell volume [fL]
J
Wildtype H2B-mCitrine
YPD SCD SCGE
Rel. mRNA concentration norm. on WT
HTB1 HTB2 ACT1
K
H2B-mCitrine (YPD) H2B-mCitrine (SCGE)
Autofluorescence Control
x10^4
H2B-mCitrine amount at birth [arb.units]
Time rel. to nutrient switch [min]

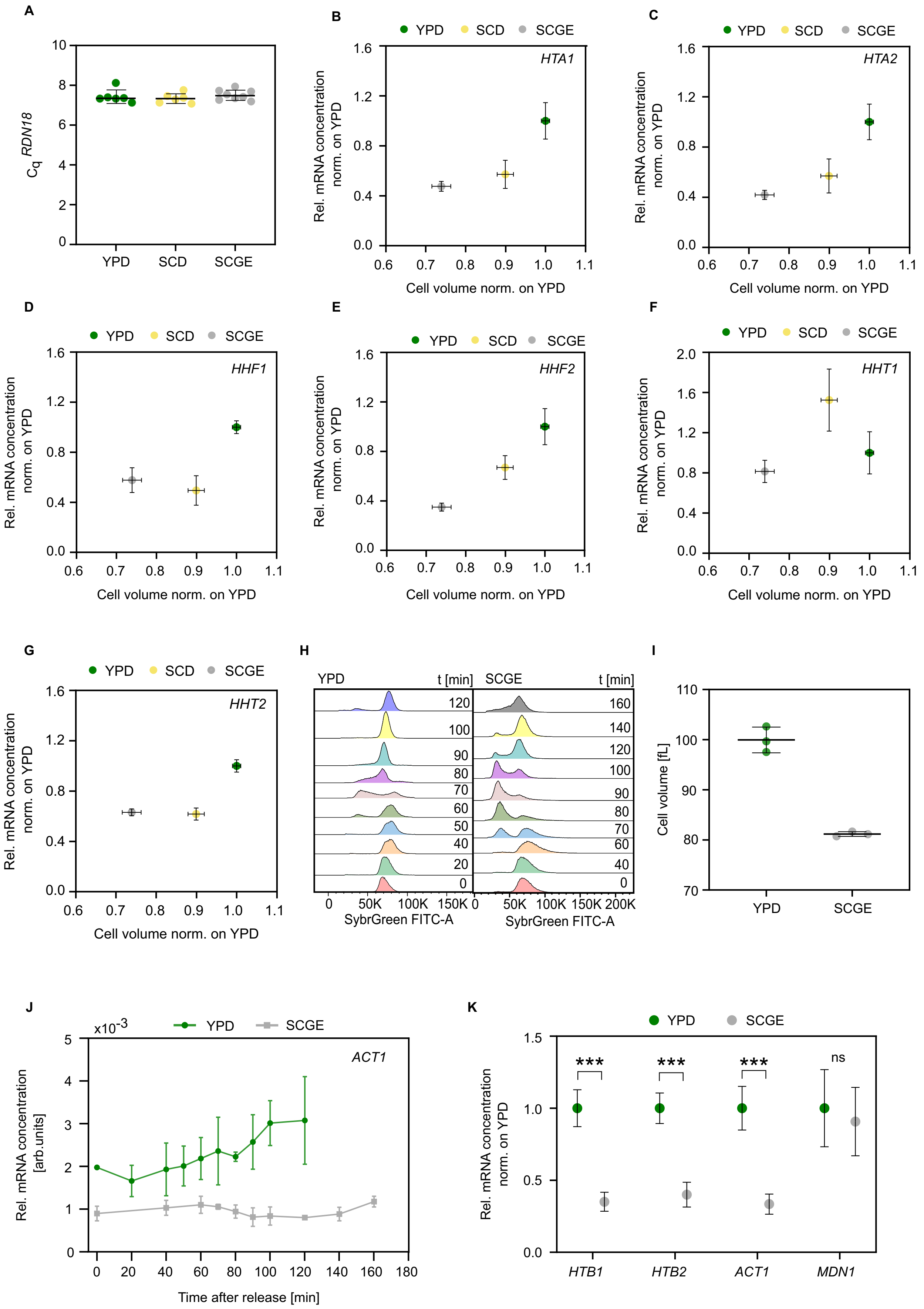
A
B
C
D
E
F
G
H
I
J
K
YPD
SCD
SCGE
Cq RDN18
Rel. mRNA concentration norm. on YPD
Cell volume norm. on YPD
HTA1
HTA2
HHF1
HHF2
HHT1
HHT2
t [min]
SybrGreen FITC-A
Cell volume [fL]
x10-3
ACT1
Rel. mRNA concentration [arb.units]
Time after release [min]
***
ns
HTB1
HTB2
MDN1

 © The Author(s)

Figure EV2. Histone mRNA concentrations decrease with decreasing nutrient-specific cell volume.

(**A**) C_q values of the reference ribosomal RNA *RDN18* were obtained by RT-qPCR analysis across different nutrient conditions. Lines and error bars represent the means and standard deviations of $n = 6$–8 independent measurements shown as individual dots. (**B–G**) Relative mRNA concentrations of *HTA1* (**B**), *HTA2* (**C**), *HHF1* (**D**), *HHF2* (**E**), *HHT1* (**F**), and *HHT2* (**G**) as a function of the relative nutrient-specific cell volume. Mean and standard deviation of at least four biological replicates are shown. (**H**) Exemplary nutrient-dependent cell cycle distributions were measured by flow cytometry at defined time points throughout the cell cycle of synchronized cells with β-estradiol-inducible *CDC20*. (**I**) Mean cell volumes of cells with β-estradiol-inducible *CDC20*, measured in YPD and SCGE at $t = 70$ min and $t = 100$ min, respectively. Lines and error bars represent the means and standard deviations of $n = 3$ independent measurements shown as individual dots. (**J**) mRNA concentrations of *ACT1* (normalized to *RDN18*) were determined using RT-qPCR after synchronous release into the cell cycle ($t = 0$) triggered by the addition of 200 nM β-estradiol. Mean and standard deviation of four biological replicates are shown. (**K**) RT-qPCR analysis of the relative mRNA concentrations of *HTB1*, *HTB2*, *ACT1*, and *MDN1* in asynchronous cells with β-estradiol-inducible *CDC20*. Mean fold changes with respect to YPD and standard deviations of $n = 3$–6 replicate measurements are shown; $^{***}P_{HTB1} = 1.35 \times 10^{-5}$; $^{***}P_{HTB2} = 2.18 \times 10^{-5}$; $^{***}P_{ACT1} = 3.08 \times 10^{-5}$.

© The Author(s)

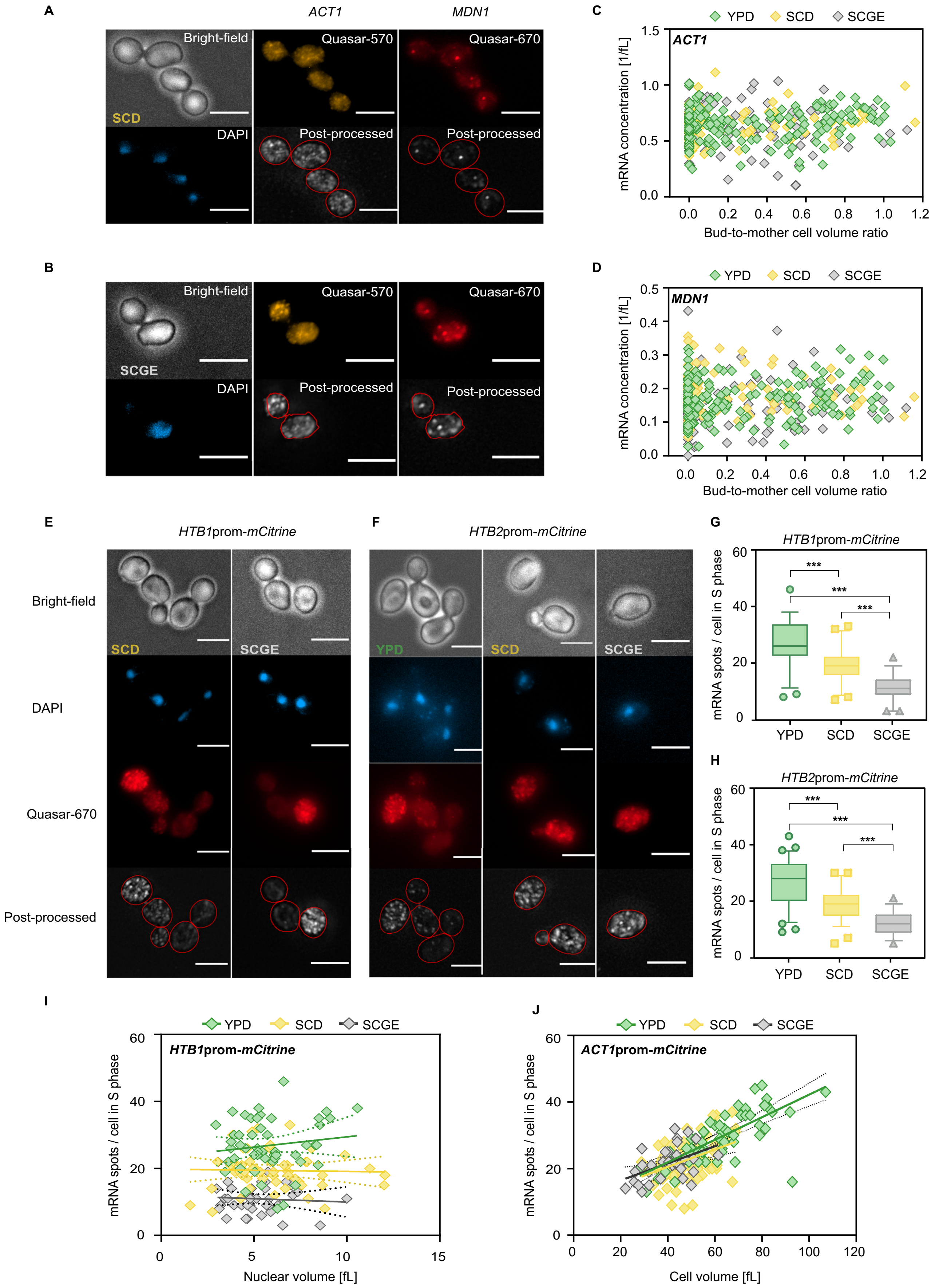
A
ACT1
MDN1
Bright-field
Quasar-570
Quasar-670
SCD
DAPI
Post-processed
Post-processed
B
Bright-field
Quasar-570
Quasar-670
SCGE
DAPI
Post-processed
Post-processed
C
YPD SCD SCGE
ACT1
mRNA concentration [1/fL]
Bud-to-mother cell volume ratio
D
YPD SCD SCGE
MDN1
mRNA concentration [1/fL]
Bud-to-mother cell volume ratio
E
HTB1prom-mCitrine
Bright-field
DAPI
Quasar-670
Post-processed
SCD
SCGE
F
HTB2prom-mCitrine
YPD
SCD
SCGE
G
HTB1prom-mCitrine
mRNA spots / cell in S phase
YPD SCD SCGE
H
HTB2prom-mCitrine
mRNA spots / cell in S phase
YPD SCD SCGE
I
YPD SCD SCGE
HTB1prom-mCitrine
mRNA spots / cell in S phase
Nuclear volume [fL]
J
YPD SCD SCGE
ACT1prom-mCitrine
mRNA spots / cell in S phase
Cell volume [fL]

 © The Author(s)

Figure EV3. smFISH analysis of cell cycle-dependent gene expression in different nutrient conditions.

ACT1 and *MDN1* transcripts were detected in single cells using smFISH probes labeled with Quasar-570 (yellow) and Quasar-670 (red), respectively. Nuclear DNA was stained with DAPI (blue). For mRNA quantification, images were post-processed as described in Methods and Materials. Representative images of cells grown in SCD (**A**) and SCGE (**B**) are shown. The scale bars represent 5 µm. (**C**, **D**) mRNA concentrations (mRNA spots per cell volume) of (**C**) *ACT1* ($n_{YPD} = 176$, $n_{SCD} = 87$, $n_{SCGE} = 98$) and (**D**) *MDN1* ($n_{YPD} = 176$, $n_{SCD} = 87$, $n_{SCGE} = 98$) were plotted against the corresponding bud-to-mother cell volume ratio in different nutrients. (**E**, **F**) Representative images of cells expressing (**E**) *HTB1prom-mCitrine* and (**F**) *HTB2prom-mCitrine* in different growth media. *mCitrine* transcripts were detected using probes labeled with Quasar-670 (red). Cell nuclei were stained with DAPI (blue). For mRNA quantification, images were post-processed as described in *Methods and Materials*. Scale bars represent 5 µm. (**G**, **H**) *mCitrine* mRNA amounts expressed from the (**G**) *HTB1* (*HTB1*prom-*mCitrine*; $n_{YPD} = 50$, $n_{SCD} = 51$, $n_{SCGE} = 39$) and (**H**) *HTB2* promoter (*HTB2*prom-*mCitrine*; $n_{YPD} = 64$, $n_{SCD} = 59$, $n_{SCGE} = 50$) in S-phase, as quantified by smFISH. Box plots represent median and 25th and 75th percentiles, whiskers indicate the 5th and 95th percentiles and symbols show outliers; *HTB1prom-mCitrine* (***$P_{YPD\text{-}SCGE} = 5.29 \times 10^{-20}$; ***$P_{YPD\text{-}SCD} = 4.07 \times 10^{-7}$; ***$P_{SCD\text{-}SCGE} = 2.31 \times 10^{-11}$); *HTB2prom-mCitrine* (***$P_{YPD\text{-}SCGE} = 5.42 \times 10^{-23}$; ***$P_{YPD\text{-}SCD} = 1.19 \times 10^{-8}$; ***$P_{SCD\text{-}SCGE} = 4.63 \times 10^{-11}$). (**I**) Number of *HTB1*prom-*mCitrine* mRNA spots per cell ($n_{YPD} = 50$, $n_{SCD} = 51$, $n_{SCGE} = 39$) as a function of nuclear volume during S-phase. Here, cells with a bud-to-mother volume ratio <0.3 were considered to be in S-phase. Lines show linear fits; dashed lines indicate the 95% confidence intervals. (**J**) Number of *ACT1* promoter-*mCitrine* mRNA spots per cell ($n_{YPD} = 59$, $n_{SCD} = 75$, $n_{SCGE} = 33$) as a function of cell volume during S-phase. Here, cells with a bud-to-mother volume ratio <0.3 were considered to be in S-phase. Lines show linear fits; dashed lines indicate the 95% confidence intervals.

© The Author(s)

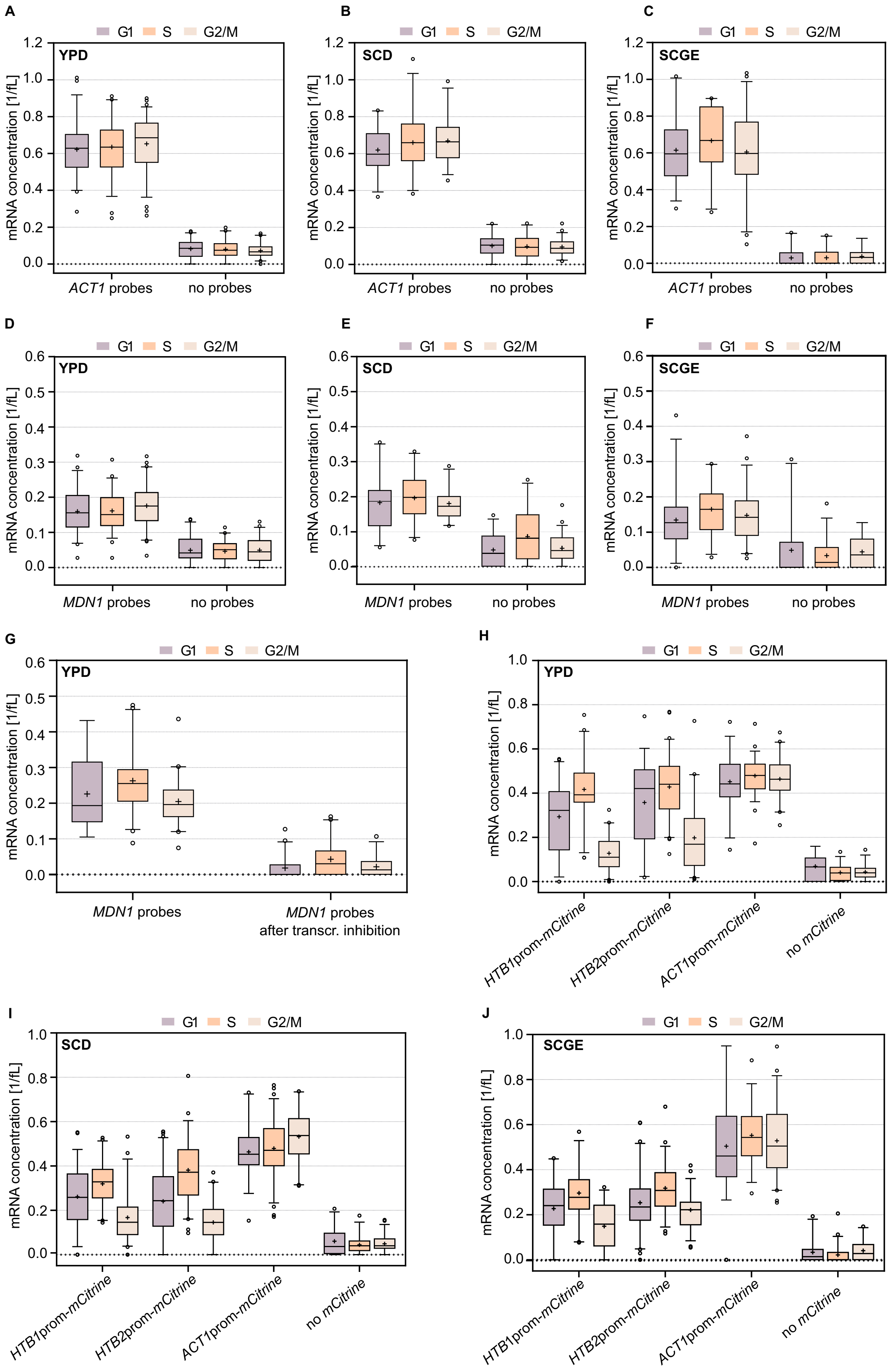
A
B
C
D
E
F
G
H
I
J
G1 S G2/M
YPD
SCD
SCGE
mRNA concentration [1/fL]
ACT1 probes
no probes
MDN1 probes
MDN1 probes after transcr. inhibition
HTB1prom-mCitrine
HTB2prom-mCitrine
ACT1prom-mCitrine
no mCitrine

 © The Author(s)

Figure EV4. mRNA concentration of transcripts of interest in G1-, S- and G2/M-phase measured with smFISH are shown in comparison to negative controls.

(**A–F**) Wild-type cells were incubated with or without smFISH probes against *ACT1* (**A–C**) or *MDN1* (**D–F**), *ACT1, MDN1* ($n_{G1}^{YPD} = 48$, $n_{S}^{YPD} = 54$, $n_{G2M}^{YPD} = 74$, $n_{G1}^{SCD} = 26$, $n_{S}^{SCD} = 31$, $n_{G2M}^{SCD} = 30$, $n_{G1}^{SCGE} = 26$, $n_{S}^{SCGE} = 23$, and $n_{G2M}^{SCGE} = 49$); no probes ($n_{G1}^{YPD} = 49$, $n_{S}^{YPD} = 40$, $n_{G2M}^{YPD} = 43$, $n_{G1}^{SCD} = 28$, $n_{S}^{SCD} = 27$, $n_{G2M}^{SCD} = 52$, $n_{G1}^{SCGE} = 24$, $n_{S}^{SCGE} = 30$, and $n_{G2M}^{SCGE} = 16$). (**G**) Wild-type cells were treated with thiolutin for 80 min to inhibit global transcription prior to incubation with *MDN1* probes. mRNA concentrations in G1, S, and G2/M were estimated by dividing the number of detected spots by the cell volume ($n_{G1}^{MDN1} = 14$, $n_{S}^{MDN1} = 42$, $n_{G2M}^{MDN1} = 40$, $n_{G1}^{MDN1,aftertranscr.inhibition} = 57$, $n_{S}^{MDN1,aftertranscr.inhibition} = 40$, and $n_{G2M}^{MDN1,aftertranscr.inhibition} = 34$). (**H–J**) Wild-type cells expressing no mCitrine, as well as cells carrying an additional copy of the *HTB1, HTB2*, or *ACT1* promoter driving mCitrine were incubated with smFISH probes against *mCitrine*. mRNA concentrations in G1, S, and G2/M were estimated by dividing the number of detected spots by the cell volume. Box plots represent median and 25th and 75th percentiles; whiskers indicate the 5th and 95th percentiles and symbols show outliers; *HTB1prom-mCitrine* ($n_{G1}^{YPD} = 58$, $n_{S}^{YPD} = 49$, $n_{G2M}^{YPD} = 41$, $n_{G1}^{SCD} = 54$, $n_{S}^{SCD} = 51$, $n_{G2M}^{SCD} = 53$, $n_{G1}^{SCGE} = 28$, $n_{S}^{SCGE} = 39$, and $n_{G2M}^{SCGE} = 28$); *HTB2prom-mCitrine* ($n_{G1}^{YPD} = 37$, $n_{S}^{YPD} = 64$, $n_{G2M}^{YPD} = 59$, $n_{G1}^{SCD} = 85$, $n_{S}^{SCD} = 59$, $n_{G2M}^{SCD} = 50$, $n_{G1}^{SCGE} = 65$, $n_{S}^{SCGE} = 50$, and $n_{G2M}^{SCGE} = 55$); *ACT1prom-mCitrine* ($n_{G1}^{YPD} = 35$, $n_{S}^{YPD} = 59$, $n_{G2M}^{YPD} = 41$, $n_{G1}^{SCD} = 38$, $n_{S}^{SCD} = 75$, $n_{G2M}^{SCD} = 42$, $n_{G1}^{SCGE} = 37$, $n_{S}^{SCGE} = 33$, and $n_{G2M}^{SCGE} = 54$); *no mCitrine* ($n_{G1}^{YPD} = 15$, $n_{S}^{YPD} = 28$, $n_{G2M}^{YPD} = 37$, $n_{G1}^{SCD} = 32$, $n_{S}^{SCD} = 30$, $n_{G2M}^{SCD} = 48$, $n_{G1}^{SCGE} = 26$, $n_{S}^{SCGE} = 49$, and $n_{G2M}^{SCGE} = 31$).

© The Author(s)

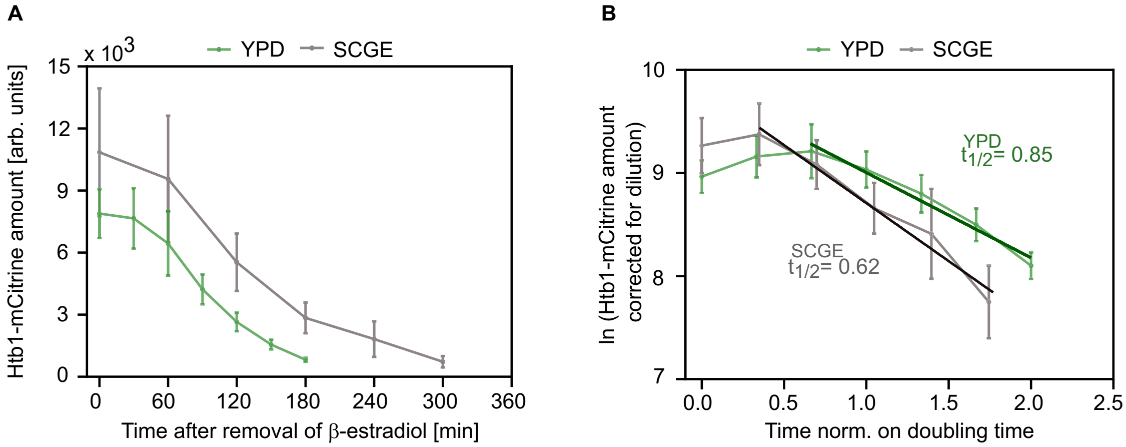

Figure EV5. Analysis of nutrient-dependent histone protein degradation suggests that histone stability does not compensate for the transcriptional downregulation in poor growth media.

To study the degradation of histone proteins in different nutrients, we constructed a strain carrying an extra copy of *HTB1-mCitrine* expressed from a β-estradiol-inducible promoter. First, Htb1-mCitrine was expressed by the addition of 10 nM or 7 nM β-estradiol to YPD or SCGE, respectively. Upon removal of the hormone from the growth media, protein synthesis was inhibited, and the degradation of Htb1-mCitrine in asynchronous cell cultures was monitored over time using flow cytometry. (**A**) Htb1-mCitrine protein degradation curves in YPD and SCGE measured after β-estradiol removal. For each time point, the mean and standard deviation of three biological replicates is plotted. (**B**) Nutrient-specific protein half-lives were obtained from linear regression on the natural logarithm of the mCitrine amounts as a function of time normalized to the respective doubling time. The mCitrine amounts were corrected to account for the dilution through cell division. For each time point, the mean and standard deviation of three biological replicates is shown.

 © The Author(s)
